# Natural Products as Modulators of Sirtuins

**DOI:** 10.3390/molecules25143287

**Published:** 2020-07-20

**Authors:** Berin Karaman Mayack, Wolfgang Sippl, Fidele Ntie-Kang

**Affiliations:** 1 Department of Pharmaceutical Chemistry, Faculty of Pharmacy, Istanbul University, Istanbul 34116, Turkey; 2 Institute of Pharmacy, Martin-Luther University of Halle-Wittenberg, Kurt-Mothes-Str. 3, 06120 Halle (Saale), Germany; wolfgang.sippl@pharmazie.uni-halle.de (W.S.); ntiekfidele@gmail.com (F.N.-K.); 3 Department of Chemistry, University of Buea, P.O. Box 63, Buea CM-00237, Cameroon; 4 Institute of Botany, Technical University of Dresden, 01217 Dresden, Germany

**Keywords:** natural products, sirtuin, drug discovery, epigenetics, structure–activity relationship

## Abstract

Natural products have been used for the treatment of human diseases since ancient history. Over time, due to the lack of precise tools and techniques for the separation, purification, and structural elucidation of active constituents in natural resources there has been a decline in financial support and efforts in characterization of natural products. Advances in the design of chemical compounds and the understanding of their functions is of pharmacological importance for the biomedical field. However, natural products regained attention as sources of novel drug candidates upon recent developments and progress in technology. Natural compounds were shown to bear an inherent ability to bind to biomacromolecules and cover an unparalleled chemical space in comparison to most libraries used for high-throughput screening. Thus, natural products hold a great potential for the drug discovery of new scaffolds for therapeutic targets such as sirtuins. Sirtuins are Class III histone deacetylases that have been linked to many diseases such as Parkinson`s disease, Alzheimer’s disease, type II diabetes, and cancer linked to aging. In this review, we examine the revitalization of interest in natural products for drug discovery and discuss natural product modulators of sirtuins that could serve as a starting point for the development of isoform selective and highly potent drug-like compounds, as well as the potential application of naturally occurring sirtuin inhibitors in human health and those in clinical trials.

## 1. Introduction

Today, the journey of a drug, starting from the discovery of the first-compound active in vitro to the shelves in a drug store, takes about 13 years and costs over 1.5 billion dollars [1]. The increase in the cycle time and the total cost of a drug discovery project starting from hit identification to pharmaceutical manufacturing have mainly increased due to the efforts of searching for new treatments for rather challenging diseases, increase in safety requirements associated with animal models and particularly with clinical trials [1]. Higher attrition rates in the late stages of the drug discovery pipeline especially in Phase II and Phase III clinical trials have also placed substantial stress on pharmaceutical and biotech companies [1,2,3]. In addition, when the Human Genome Project was completed in 2003, the pharmaceutical industry faced another productivity challenge as a significant number of targets with therapeutic relevance were revealed [4,5]. In return, a very rewarding with higher scientific risks and competitive era has started for the pharmaceutical industry with the post-genome area [6].

Although, there were many targets with no known small molecule modulators, pharmaceutical companies needed to establish new molecular entities with a potential intellectual property. This competition resulted in establishments of translational and chemical screening programs both at industrial and academic level [7,8]. Moreover, rapid advances in combinatorial chemistry accelerated the much-needed growth in the size of screening libraries where a large number of compounds can be prepared in a single process by combining different chemical synthetic methods and few starting materials [9]. The rapid increase in the number of screening compounds raised the need for a method that would be able to find biologically relevant compounds from a large set of molecules. In order to do that in a time-efficient way, automated high-throughput screening (HTS) facilities were built [10,11]. HTS and did not need prior information about the target and the ligands [6]. 

However, it did not take so long for the pharmaceutical industry to realize that only a few drugs derived from HTS campaigns could make it to the market [12]. One of the main reasons behind the poor productivity rates of HTS was the composition of the libraries [6,8]. Although there were problems with the size of the screening libraries, the main limitation was the quality of the compounds. In comparison to alternative approaches such as fragment-based drug discovery, hits with less desirable physicochemical properties could be derived from HTS studies [13]. Moreover, pharmaceutical companies realized that chemical space covered in combinatorial libraries were not able to meet the rich structural diversity of newly discovered enzymes nor the protein–protein interactions [14]. In order to generate HTS compound libraries that could reflect the portfolio of novel target classes, companies started to clean up their libraries from unwanted compounds and include new chemotypes, Thus, the library design focused mainly on compounds that show drug-like or lead-like properties [15,16]. In addition, retrospective analysis of HTS data by Novartis revealed that natural products (NPs) represent the most diverse compound class and plates containing NPs would significantly increase the chance of identifying a hit molecule compared to the traditional synthetic and combinatorial chemistry compound collections [17]. Companies started including more atypical chemotypes such as NP mimics in their HTS collections.

A detailed analysis of all therapeutic agents approved over the time frame between 1981 and 2014 revealed that 64% of the new therapeutics were NPs, or NP derivatives, or synthetic drugs with a NP pharmacophore or compounds that mimic a NP [12]. Especially, in the area of cancer, almost half of the small molecules that were approved between the 1940s and 2014 were either NPs or NP derivatives. Drugs such as lovastatin, paclitaxel, silibinin, and penicillin were derived either directly or indirectly from NPs [18,19]. These statistical analyses revealed that “Natural products have a great potential to search for novel lead structures for drug discovery and development purposes.”

Historically, NPs represented a vital source of all medical treatments. However, there has been a significant decrease in the NPs used for drug discovery in the pharmaceutical industry [20,21]. The legitimate concerns to screen for NPs in HTS campaigns partly arose from the regulations accepted under the United Nations Convention on Biological Diversity and the Nagoya Protocol [22]. Moreover, there was a belief in the pharmaceutical industry that NPs were not compatible with the assays implemented in HTS directed at macromolecular targets [23]. Another concern about using natural products in drug discovery approaches was related with the technical problems such as purification, structure elucidation of the active ingredients from extracts, the need for repeated isolation of the active ingredient to be able to have enough for biochemical assays, and developing a synthetic method to be able to produce the active ingredient during pharmaceutical manufacture [14]. 

It is also now possible to characterize the structures of NPs with a higher sensitivity owing to the recent advances in Nuclear Magnetic Resonance (NMR) spectroscopy. One of the biggest challenge associated with the structural elucidation of NP structures was the assignment of stereochemistry to molecules. Development of J-resolved NMR technique made it possible to analyze the overlapping signals by achieving a complete separation of chemical shifts and J couplings [24]. In addition, more information about the bond distance and bond angles can be retrieved from the magnitude of the J coupling which could be then correlated with stereochemical features of the compound. Moreover, high-performance liquid chromatography coupled with mass spectrometry has been used to generate fractions of concentrated extract samples for HTS strategies [25,26]. Simplifying extracts and removing molecules that are likely to give false positives in biological testing have improved the applicability of NP screening collections for HTS campaigns [22]. Thus, a combination of pre-fraction HTS strategies and sensitivity enhancement in NMR techniques has resolved the challenging structural elucidation and isolation problems.

A new phase has already started for the identification of novel and potent NPs by introducing overexpression and antisense-suppression strategies into pharmaceutical development [27,28]. Overexpression of genes for biosynthetic enzymes that produce the metabolites of therapeutic interest in cultivable organisms/plants is a promising approach that could solve the problems related to the production of the NPs in industrial scale. An effective antimalarial drug, artemisinin, was the first successful example of the use of metabolic engineering to produce semi-synthetic NPs [29,30]. In conclusion, it is now recognized that NPs and their derivatives possess therapeutic benefits in treating diseases and have market potential. 

### 1.1. Characteristics of Natural Products and Development of Natural Product Databases for Drug Discovery 

NPs are often defined as “molecules obtained from natural sources that exhibit biological activities” [31]. Thus, the success of NPs can be explained by how they are biologically active and are designed to bind to biological macromolecules [32]. Being secondary metabolites, NPs complement the biological structural space so-called ‘protein-binding sites’ [33]. Since NP-protein interactions are already optimized in nature, NP derived scaffolds may serve as superior starting points for lead optimization than random three-dimensional (3D) structures [22,34]. Moreover, their biological compatibility provides the underlying reason why they have favorable ADMET (absorption, distribution, metabolism, excretion, and toxicity) properties. Also, their tendency to be more hydrophilic makes them likely to be substrates for transporter systems as they would need to be transferred into the cell to initiate their mechanism of action [22,33,35].

Using a variety of molecular descriptors, Dabson et al., compared the similarity of known drugs and compounds in screening collections to human metabolites [36]. Results suggested that known drugs are in general more similar to the naturally occurring metabolites than the compounds in most screening collections. These results strengthened the notion that NPs may have further benefits over medicinal chemistry compounds as they are inherently bioactive compounds. 

Interestingly, statistical analysis of the structural properties of NPs and synthetic compounds showed that NPs occupy a larger, more diverse, and more drug-like chemical space than do synthetic compounds [35,37]. Hert et al., observed that more than eighty percent of the core ring-scaffolds that are present in nature-derived products were missing in commercial libraries [38]. Occupying larger pharmacophore diversity as well as a high degree of stereochemistry, NPs represent also a good starting point for un-druggable targets. NPs such as rapamycin analogs were already shown to be valuable therapeutics against difficult screening targets like protein-protein interactions [39].

Advances in combinatorial chemistry techniques enable development of large compound collections for HTS screening for drug discovery. However, it has become evident that those collections could only cover a limited biologically relevant chemical space. On the other hand, NPs, being biologically validated, have continued to be a vital resource that provides lead compounds for clinical trials and therapeutic agents for the market. Recent technological advances have also significantly reduced the technical barriers of exploitation of NPs in HTS against molecular targets. It has become crucial to include NPs and/or NP-derived compounds in databases for both high throughput and virtual screening in drug discovery. 

Currently, a large number of publicly available or commercial NP databases have been developed such as Naturally Occurring Plant-Based Anti-cancer Compound-Activity-Target database (NPACT), Traditional Chinese Medicine Integrated Database (TCMID), Super Natural II, TCM Database@Taaiwan, Northern African Natural Products Database (NANPDB), Nuclei of Bioassays, Ecophysiology and Biosynthesis of Natural Products Database (NuBBE_DB_) etc. These databases provide specific information about NPs regarding their physicochemical and structural features, related target proteins, pharmacological activities, predicted toxicity profile, vendor information, and so on. However, the availability of natural compound libraries exploring the biodiversity of different regions around the World is very limited and a vast majority of the living species globally has not been systematically investigated for drug discovery purposes. Moreover, the relevance of medicinal plants or herbs commonly used by different cultures as part of their traditional medicine needs to be explored more thoroughly.

### 1.2. Sirtuins as Therapeutic Targets

Over the last twenty years, histone deacetylases (HDACs) attracted much attention giving their regulatory roles in many pathophysiological conditions. HDACs regulatory mechanism on gene expression depends on the removal of the acetyl group from ε-amino group of acetylated lysine residues located in histone terminal tails. This in return causes a condensed and inactive chromatin structure and as a result gene silencing. In addition, some HDAC isoforms have been shown to act as well on non-histone proteins and are able to remove other lysine-modifications.

HDACs are classified in four main groups. While Class I, II, and IV are dependent on a Zn^2+^ ion as a cofactor to catalyze the deacetylation reaction, Class III HDACs use nicotinamide adenine dinucleotide (NAD^+^) for the removal acetyl moiety on lysine residues. Seven sirtuin isoforms have been identified in humans (Sirt1–7) which differ in their subcellular localization, as a result their substrate preferences and interacting partners and thus, their role in cellular processes. It has been shown that sirtuins not only deacetylate histones but also certain non-histone proteins. Moreover, sirtuins are also shown to hydrolyze structurally diverse acyl groups and efficiently catalyze different post-translational modifications such as e.g., decrotonylation, demalonylation, desuccinylation, demyristoylation, depropionylation, and delipoamidation [40].

In order to understand the catalytic mechanism of sirtuins and their interaction with different substrates and partners and also their modulation in the existence of structurally diverse inhibitors and activators, several X-ray crystal structures have been determined. A detailed review on sirtuin structures and substrates can be found in elsewhere and the reader is referred to these recent reviews [41]. Overall, sirtuins share a highly conserved catalytic core of 275 amino acids, which is composed of two globular subdomains—a large Rossmann-fold domain and a smaller and structurally more diverse zinc-binding domain. These two subdomains are connected by four flanking loops forming the large active cleft where cofactor NAD^+^ and the substrate bind in reverse directions. It is important to note that, although all sirtuins share a highly conserved catalytic pocket, they are distinct in terms of length, amino acid composition, and flexibility of their N- and C- termini. Varieties in their terminal segments were suggested to impact sirtuins’ functional activities as well as recognition of other proteins and interactions with molecular partners [42].

As sirtuins target a vast number of non-histone proteins, their involvement in the regulation of a wide range of physiological and pathological processes is inevitable. Sirtuins have been reported to involve in the pathogenesis of many diseases such as cancer, HIV, metabolic and neurodegenerative disorders, and parasitic diseases [43]. Sir2 gene was found to regulate the cell survival and a longer life span in calorie restriction [44], this discovery triggered extensive research on sirtuin proteins and their value as therapeutic targets. For example, neuroprotective role of Sirt1 in Huntington’s disease and Alzheimer’s disease have been shown [45,46,47]. In contrary, inhibition or knockdown of Sirt2 has shown to be neuroprotective against Huntington’s disease and Parkinson’s disease [48,49]. Sirt1 and 2 are not the only isoforms of sirtuins that have regulatory roles in neurons. Pfister et al., reported that overexpression of Sirt3 and Sirt6 is neurotoxic in neurons, on the other hand, subcellular localization of Sirt5 has an effect on neuronal viability. When Sirt5 is localized in cytoplasm, it played a neuroprotective role. However, when this protein is localized in the mitochondria, it induced apoptosis [50]. 

Similar to their opposing effects in neurodegenerative diseases and on neuronal survival, sirtuins also employ inconsistent activities in response to cancer. Sirt1 was first recognized as an oncogene, as overexpression of Sirt1 could repress expression and/or activity of several tumor suppressor genes and proteins that are involved in DNA repair [51]. Upregulation of Sirt1 expression in many cancer types such as breast cancers, leukemia, prostate, and colon cancers also support the tumor promoter role of Sirt1 [52,53,54]. In contrast, Sirt1 levels were decreased in various cancer tissues including bladder, prostate and ovarian cancers, implying that Sirt1 might be a tumor suppressor [55]. Moreover, combination of Sirt1 inhibitor, Ex527, with an HDAC inhibitor, valproic acid, or butyrate, induced apoptosis in human leukemia cells [56]. Ghosh et al., also demonstrated that a novel quinoxaline based small molecule inhibitor of Sirt1 induced apoptosis of colon cancer cells [57]. Similar to Sirt1, the data related to the role of Sirt2 in cell proliferation and development of cancer are controversial. Down regulation of Sirt2 levels in numerous cancer types such as gastric carcinoma, non-small lung cancer types and glioma imply that Sirt2 might be a tumor suppressor. In concordance with these results, Sirt2 inhibition with small molecules such as Sirtinol, Cambinol, AC-93253, etc., has shown to be useful in treating many cancer cell lines including prostate, pancreas, breast, oral, and lung cancer cell lines [58]. It is also not clear whether mitochondrial sirtuin deacetylase Sirt3 is a tumor suppressor or a tumor promoter or is it dependent on cell or tumor type [59]. Kim et al., showed in their studies that Sirt3 deficient mouse embryonic fibroblasts became immortalized when infected with RAS or MYC oncogenes. Moreover, authors also demonstrated that Sirt3 expression levels were decreased in several cancer types such as breast, testicular, prostate, head, and neck cancers [60]. However, another study showed that Sirt3 acts as promoter of cell proliferation and survival in oral cancer carcinogenesis and that down-regulation of Sirt3 increased sensitivity of oral squamous cell carcinoma cell lines to radiation and cisplatin treatments [61]. Meanwhile, Sirt4 has shown to be downregulated in many cancer types and appears to modulate tumor cell metabolism [62] and adopts a tumor suppressor role colorectal cancer [63], Myc-induced B cell lymphoma [64] and also in esophageal squamous cell carcinoma [65]. Like Sirt1-3, Sirt5 was shown to have dual tumor suppressor and tumor promoter activities [66]. While Sirt5 was found to be overexpressed in human non–small cell lung cancer [67], Sirt5 levels were considerably downregulated in head and neck squamous cell carcinoma [68]. Another sirtuin isoform which shows tissue specific functions in tumorigenesis is Sirt6. While Sirt6 adopted a tumor suppressor role in pancreatic [69], breast and colon cancers [70], and hepatocellular carcinoma [71], a tumor promoter role was played by this protein in other cancer types such as lung, prostate, and melanoma [72].

Sirtuins are not only involved in neuronal and non-neuronal cell viability but they also regulate immune and inflammatory responses, metabolism, aging and readers are referred to the articles that reviewed sirtuin biology in these respects [73,74,75,76,77,78,79,80]. Collectively, these studies show that sirtuins are linked to the pathogenesis of many age-related diseases and represent an interesting class of epigenetic proteins that bear a great potential as drug targets. 

## 2. Natural Product Modulators of Sirtuins

In order to validate sirtuins as therapeutic targets, compounds that are isoform selective, potent, and cell-permeable with suitable pharmacokinetic properties need to be identified. Using high-throughput or in silico based screening approaches or fragment-based drug discovery experiments, several structurally diverse molecules have been found with either activation or inhibition properties against sirtuins. To name a few, nicotinamide and its analogs, analogs of cofactor NAD^+^, indoles, derivatives of hydroxynaphthaldehyde, pseudopeptides, or substrate mimics, are present among the list [41]. Recently, several compounds with promising potency, isoform selectivity and drug-like properties have been identified. Sirtuin Rearranging Ligands (SirReals) are one class of inhibitors that fulfill these attributes [81]. Although, there have been many attempts to discover the therapeutic potential of Sirtuins with small molecule inhibitors, there is still no drug that approved for clinical use. So far, Selisistat, a Sirt1 inhibitor, remains to be the only compound that could reach clinical trials [82,83]. In this chapter, we will focus on NP modulators of sirtuins with promising biological activities.

### 2.1. Stilbenoids: Resveratrol, Piceatannol, and Trans-(−)-ϵ-Viniferin

Resveratrol, a member of natural polyphenols, is found in considerable amounts in plants such as grapes, blueberries, cranberries, as well as peanuts, groundnuts and Japanese knotweed [84]. Therapeutic value of resveratrol has been studied extensively in the last decade revealing its remarkable biological activities including antioxidant, anti-inflammatory, anticancer, and cardio- and neuroprotective effects [85,86,87,88,89]. Resveratrol was the first polyphenol proposed to be a direct activator of Sirt1 [90]. Later on a weak inhibitory activity of resveratrol against the cytoplasmic sirtuin, Sirt2, and the mitochondrial sirtuin, Sirt3, was shown and similar to Sirt1, resveratrol could stimulate Sirt5 activity [90,91,92]. Moreover, poor bioavailability of this compound did not allow reach high plasma levels after oral administration, raising the question whether there exists any another mechanism that mediates the indirect Sirt1 activation [93]. New evidences indicate that 5′ adenosine monophosphate-activated protein kinase (AMPK), one of the key regulators of cellular energy homeostasis, mediate indirect activation of Sirt1 by resveratrol. It was suggested that AMPK induces Sirt1 activity by increasing the NAD^+^ levels and that this two enzymes are interdependent for metabolic adaptation [94,95]. Similarly, another stilbenoid, trans-(−)-ϵ-Viniferin was shown to increase mitochondrial Sirt3 levels and have an indirect impact on Sirt3 deacetylase activity [96]. Viniferin was shown to activate AMPK which in turn would increase the cellular NAD^+^ levels and the increased NAD^+^ levels would activate Sirt3-dependent deacetylation [96].

**Figure 1:**
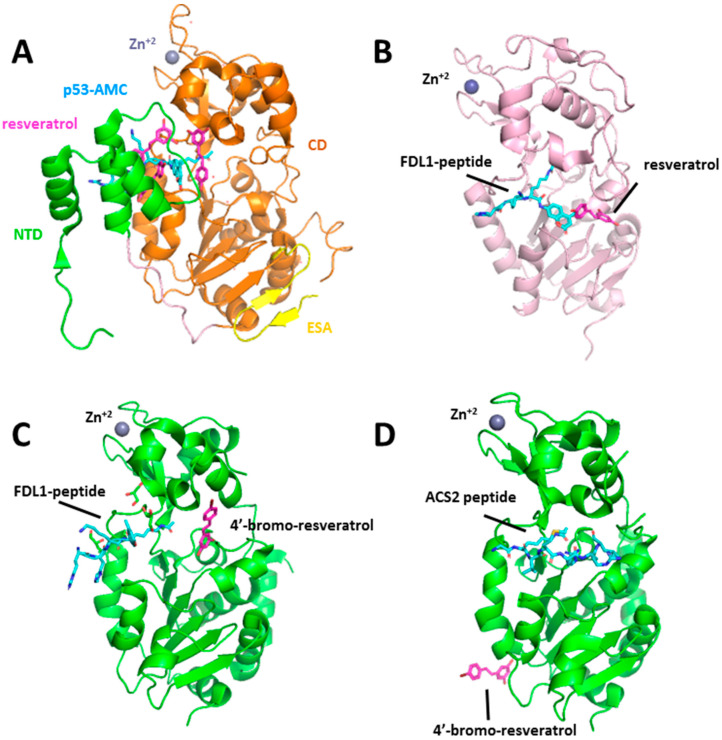
Crystal structure of human sirtuins in complex with resveratrol and 4’-bromo-resveratrol. (**A**) The overall structure of sirtuin (Sirt)1/p53-7-amino-4-methylcoumarin (AMC)/resveratrol complex. N-terminal domain (NTD), catalytic domain (CD), and C terminal essential for Sirt1 activity (ESA) region are color-coded as in Figure 1A. (**B**) Overall structure of the Sirt5/FdL-1/resveratrol complex. (**C**) Overall structure of Sirt3/FdL-1/4′-bromo-resveratrol complex. (**D**) The overall structure of the hSirt3/ACS2/carba-NAD^+^ complex. The acetylated p53-AMC, FdL1-peptide and ACS2-peptide are shown in cyan sticks. Resveratrol and 4′-bromo-resveratrol molecules are represented as pink sticks. The zinc ion is shown as a deep blue sphere.

In addition, both resveratrol and other non-resveratrol activators of Sirt1 were shown to enhance deacetylation of fluorescent substrates but not unmodified peptides or the native peptide substrates and claimed that these compounds are not direct activators of Sirt1 [97,98,99]. X-ray crystallography was used to determine the structural basis of substrate-dependent activation of Sirt1 by resveratrol [100]. An X-ray crystal structure of Sirt1 in complex with three resveratrol molecules and a 7-amino-4-methylcoumarin (AMC)-containing peptide could be solved. Interestingly, two of the resveratrol molecules were bound to the N-terminal domain (NTD) and were making hydrogen bond interactions with both NTD and p53-AMC. On the other hand, the third resveratrol molecule was in contact with catalytic (CD) domain and the peptide. It was suggested that NTD-bound two resveratrol molecules promote a tighter binding between Sirt1 and AMC peptide, and thus stimulate Sirt1 activity (Figure 1A). The structural and functional analysis confirmed that resveratrol could only stimulate Sirt1 in the presence of an NTD in addition to the CD.

Studies on how resveratrol affects different isoforms of sirtuin have shed light on whether the sirtuin activity is dependent on the presence of a fluorophore moiety on the peptide substrate. Gertz et al., performed both in vitro and crystallographic experiments to understand the molecular mechanism behind the differential activity of resveratrol and resveratrol-like compounds against Sirt3 and Sirt5. In the case of Sirt5, effects of resveratrol were dependent on the type of the acyl modification and the length of the substrate peptide [92]. Sirt5’s deacylation activity against the succinylated forms of a fluorophore-labelled peptide substrate (FDL1-peptide) and Prx-Lys197 were inhibited by resveratrol. However, resveratrol could stimulate Sirt5-dependent deacetylation of both these fluorogenic substrates but also non-modified peptides or protein substrates such as Prx-Lys197 and Cytochrome c. Interestingly, resveratrol did not affect the deacetylation of a peptide, p53-Lys382 (RHK (acylK) LMFK), which carries a fluorophore in the N-terminal sequences instead of C-terminal side. Consistently, others have found similar resveratrol mediated activation of sirtuins against longer peptides or full substrate proteins without fluorophore [101,102]. Crystal structure of human Sirt5 was solved in complex with FDL1-peptide and resveratrol to rationalize the structure-activity relationship [92]. A direct interaction between the FdL1 substrate fluorophore and resveratrol as well as resveratrol and α2/α3 and β8/α13 loops located around the catalytic binding pocket were revealed by this complex. It was speculated that binding of resveratrol between these loops mediates the closure of the peptide channel entrance and arrangement of the interface for a more suitable substrate binding mode (Figure 1B). 

A resveratrol metabolite, piceatannol, could also stimulate Sirt5 activity, however, similar to resveratrol, it inhibited deacetylation activity of Sirt3 against fluorophore-modified peptide substrates, FdL1 and FdL2 [92]. Sirt3 X-ray structure in complex with piceatonnol and FDL-1 peptide revealed a direct interaction between the compound and the C-terminal peptide fluorophore, coumarin moiety. This interaction was suggested to influence the binding mode of the substrate, resulting in a non-productive Sirt3/substrate/inhibitor complex. Using ELISA assay, authors could also show that resveratrol has a weak inhibitory effect on Sirt3 deacetylation activity for also a non-fluorophore labeled substrate, glutamate dehydrogenase. Crystal structures of Sirt3 were also solved with a derivative of resveratrol, 4′-bromo-resveratrol, which showed a more potent inhibitory effect on Sirt3 activity than resveratrol (almost a full inhibition with a 0.2 mM compound concentration) [103]. A comparison of Sirt3 complexes in complex with the two inhibitors, 4′-bromo-resveratrol and piceatannol, and the fluorogenic peptide confirmed a different inhibition mechanism for this resveratrol analog. While piceatannol induces a non-productive binding mode of FDL-1 peptide as a result of extensive contact with the fluorophore moiety, 4′-bromo-resveratrol occupies the C-pocket and prevents the insertion of the nicotinamide moiety of the NAD^+^ but does not interact with the peptide substrate directly (Figure 1C). On the other hand, Sirt3 isoform was also solved with a fluorophore-free physiological target of Sirt3, acetyl-CoA synthetase2 (ACS2), in the presence of 4′-bromo-resveratrol (Figure 1D). This complex and competition experiments revealed that 4′-bromo-resveratrol binds to the catalytic pocket and when ACS2 peptide is bound, it can no longer bind to the inhibitory site as shown in Sirt3/FdL-1/4′-bromo-resveratrol structure.

In summary, these results suggested that resveratrol or resveratrol-like compounds can directly affect Sirtuin-dependent modulation and the fluorophore label is not required for the stimulatory effect. Moreover, small changes in the chemical structure of resveratrol-like compounds or the modification of peptide sequences might force sirtuins to adopt different conformations, and hence cause a different biological response. 

Due to its low bioavailability and solubility, resveratrol was reformulated (SRT501) by Sirtris Pharmaceuticals (Cambridge, MA, USA). However, it was reported that serious adverse effects occurred during a Phase IIa study for advanced multiple myeloma in both treatment groups receiving SRT501 monotherapy and SRT501 + bortezomib [104]. GlaxoSmithKline (GSK, https://www.gsk.com/), who took Sirtris over, suspended the study and it was concluded that SRT501 ‘may only offer minimal efficacy while having a potential to indirectly exacerbate a renal complication common in this patient population’ [104]. However, recent studies provide invaluable insights into the binding sites and the inhibition or activation mechanism for resveratrol-like compounds. These findings serve as a promising starting point for the development of resveratrol analogs or search of novel ligands with improved potency and selectivity for sirtuin isoforms.

### 2.2. Chromone-Derived Natural Products

Chromenone-derived compounds which include chromones (flavone and isoflavone) and coumarins have also been extensively studied for their numerous pharmacological activities such as anti-inflammatory [105], antioxidant anticancer [106], neuroprotective [107], antimicrobial, and anti-obesity [108]. This group of compounds also represent one of the most promising class of NPs that show diverse modulatory activities on sirtuin subtypes.

**Figure 2:**
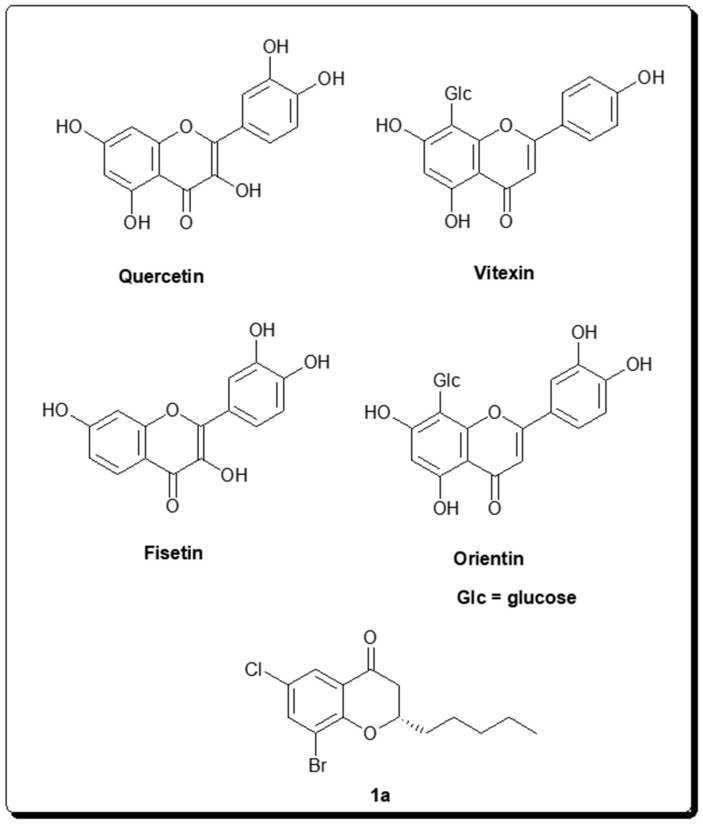
Chemical structures of selected chromenone-derived natural products as sirtuin inhibitors.

Emerging studies have demonstrated that calorie restriction may slow down aging and increase the maximum life span. One such study has spurred great interest where effects of several natural compounds were tested on Sir2 and *Saccharomyces cerevisiae* lifespan [90]. Among those compounds butein, fisetin and resveratrol significantly extended both median and maximum lifespan of *S. cerevisiae*. Moreover, the two flavones which share the same chemical structure except for a hydroxyl group in position 5, quercetin and fisetin (Figure 2), were shown to stimulate Sirt1 activity five- and seven-fold, respectively. In a more recent report, fisetin was also shown to increase average and maximum lifespan of vertebrate, mice, besides *S. cerevisiae* [109]. In addition, authors demonstrated that out of ten flavonoids (resveratrol, luteolin, rutin, curcumin, pirfenidone, myricetin, apigenin, catechin, quercetin, and epigallocatechin gallate), fisetin (Figure 2) reduced senescent markers most effectively in both primary murine embryonic fibroblasts and human fibroblasts induced to senescence. 

In another study, a series of substituted chromone/chroman-4-one derivatives with selective inhibitory effects on Sirt2 isoform were obtained by applying a one-step synthetic procedure that utilizes a microwave-assisted base-mediated aldol condensation [110]. Compounds that were substituted in the 2-, 6- and 8- positions showed the most potency against Sirt2 at the low micromolar range. The most potent compound, 8-bromo-6-chloro-2-pentylchroman-4-one (**1a**) (Figure 2), demonstrated 88% inhibitory activity against Sirt2 at 200 μM concentration in a fluorescence-based assay. Compound 1a showed less than 10% inhibition against close homologs, Sirt1 and Sirt3 subtypes, at the same concentration. Using a preparative chiral HPLC, authors could separate the enantiomers of compound 1a and found out that enantiomer (−)-**1a** (IC_50_ = 1.5μM) was slightly more potent than enantiomer (+)-**1a** (IC_50_ = 4.5 μM). This study shows that chromone/chroman-4-one derivatives scaffolds represent a good starting point to identify potent and selective Sirt2 inhibitors.

In recent years, scientists put more effort to develop screening methods that are less labor-intensive, less time-consuming, and more efficient to identify bioactive compounds from complex mixtures of NPs. These so-called ‘ligand fishing’ approaches can be classified into two—on-line and off-line modes [111]. In general, a mixture of natural products is first incubated with the immobilized macromolecules such as enzymes and receptors. During this period active compounds would bind to the biomolecules and non-binding compounds would remain in the sample solution. In the next step, for the off-line mode, ligand-bound complexes will be removed to an eluent where active compounds will be separated to be analyzed using analytical instruments such as HPLC or MS. For the on-line mode, the incubated mixture of natural products will be directly analyzed using a chromatographic system and the chromatograms of both the original and incubated sample solutions will be compared. For the incubated sample, lower signals would be detected for the bioactive compounds because of affinity binding.

Application of such a bio-guided technique which took the advantage of protein-coated magnetic beads to screen medicinal plant extracts were used to identify novel inhibitors for Sirt6 [112]. Fenugreek seed extract of *Trigonella foenum-graecum* that consists of compounds such as 4-hydroxyisoleucine (4-OH-Ile), trigonelline, naringenin, quercetin, and vitexin was used for the screen. 1% of the extract could inhibit the deacetylation of H3K9Ac by Sirt6 over 50%. It was shown that only quercetin and vitexin (Figure 2) were active against Sirt6 among all the aforementioned compounds tested. Since these two active compounds both showed a lower degree of Sirt6-mediated H3K9Ac inhibition than the whole extract and also the combination of these two compounds together did not increase the inhibitory activity than the quercetin alone, it was concluded that there are other components in the fenugreek extract responsible from the inhibition of Sirt6. A similar ligand fishing approach was used to discover additional active compounds from the *Trigonella foenum-graecum* seed extract. Using Sirt6 coated magnetic beads, authors could identify orientin (Figure 2) and seventeen other compounds as Sirt6 binders [113].

Since chromenone compounds may potentially have fluorescence property, they may interfere with fluorgenic assays that use the 7-amino-4-methylcoumarin (AMC) compound. Therefore, one should be cautious about the interpretation of the activity data in such cases [114]. Wen et al., demonstrated that flavone compounds which have been reported previously as Sirt1 activators such as quercetin, luteolin, kaempferol, baicalin, rutin, naringin, and hesperidin are all non-fluorogenic compounds. However, authors showed that isoflavone compounds such as daidzein, formononetin, calycosin, and glycitein were fluorogenic and when their fluorescent signals were removed, compounds acted as weak Sirt1 and Sirt2 inhibitors instead of what was previously reported as having Sirt1 activation activity. Moreover, phytoestrogenic isoflavones such as daidzein and genistein which are found in large amounts in soy products were shown to demonstrate inhibitory effects on muscle atrophy in C2C12 myotubes through increasing Sirt1 activity and elevating Sirt1 expression levels. However, it was suggested that these two isoflavones indirectly activate Sirt1 through inducing AMPK phosphorylation in muscle cells, which in return enhances Sirt1 activity by increasing the intracellular NAD^+^/NADH ratio.

### 2.3. Structurally Diverse Natural Product Modulators of Sirtuins

#### 2.3.1. Tanikolide Dimer from the Madagascan Marine Cyanobacterium *Lyngbya majuscula*

**Figure 3:**
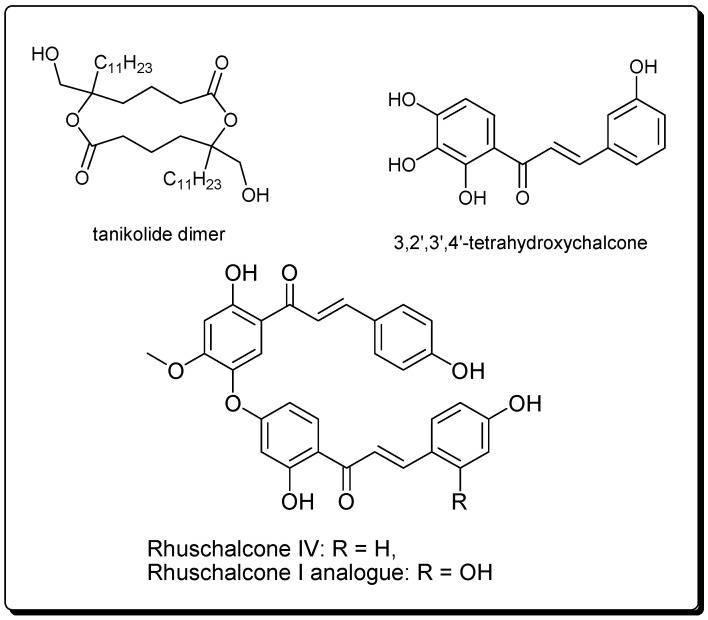
2D structure of tanikolide dimer, chalcones, and bichalcones showing sirtuin inhibitory effects.

Not only terrestrial resources but also aquatic ecosystem have been investigated for bioactive natural products, later being less prevalent. A target-based screening on marine cyanobacterial extracts led to the identification of a potent Sirt2 inhibitor, tanikolide dimer (Figure 3). The IC_50_ values of this compound varied from sub-nanomolar to low-micromolar based on the assay employed [115]. Moreover, synthetic stereoisomers of the tanikolide dimer also showed moderate inhibitory activities against Sirt1isoform and similar to native tanikolide dimer, they exhibited Sirt2 inhibition in the single-digit micromolar range [116]. 

#### 2.3.2. Bichalcones from the African Medicinal Plant *Lyngbya majuscula*

Chalcones have shown inhibitory properties against sirt1 and hindered cell growth in HEK293T cells, for example, Kahyo et al., demonstrated that 3,2′,3′,4′-tetrahydroxychalcone produces physiological effects on organisms probably through inhibiting the deacetylation by SIRT1, i.e., by inhibiting the SIRT1-mediated deacetylation of a p53 acetylated peptide and recombinant protein in vitro [117]. Besides, 3,2′,3′,4′-tetrahydroxychalcone was capable of inducing the hyperacetylation of endogenous p53, as well as increase the endogenous p21CIP1/WAF1 in intact cells, and suppress the cell growth. The authors also showed 3,2′,3′,4′-tetrahydroxychalcone to have a stronger inhibitory effect on the SIRT1-pathway than the known SIRT1-inhibitor, sirtinol. 

Bichalcones have also been shown to be potential sirtuin inhibitors [118]. Karaman et al., carried out docking of the virtual library of natural compounds from African medicinal plants with available samples (the p-ANAPL library), and tested the virtual ‘hits’ in vitro against sirtuin 1, 2, and 3 (sirt1–3) [118]. This led to the identification of two bichalcones previously isolated from the medicinal plant *Rhus pyroides* Burch (Anacardiaceae), [119,120] i.e., rhuschalcone IV and an analogue of rhuschalcone I (Figure 3). These compounds were shown to be active in the in vitro assay, with the best activity against sirt1 being an IC_50_ value of 40.8 µM for the rhuschalcone I analogue. Although several known O-linked and C-C coupled bichalcones, and biflavonoids from this plant were not tested in the assays, [121,122,123,124] research of novel more potent and selective bichalcones as sirtuin inhibitors lies in the fact that their total synthesis has been described [121,122,123,124]. It must, however, be mentioned that this class of compounds has shown diverse biological activities, e.g., cytotoxic, antiprotozoal [121,122], carbonic anhydrase inhibitory activities [123], as well as an affinity for the gamma-aminobutyric acid A (GABA_A_) benzodiazepine receptor [124].

#### 2.3.3. Flavonoids, Alkaloids, and Xanthones from Indonesian Medicinal Plants

**Figure 4:**
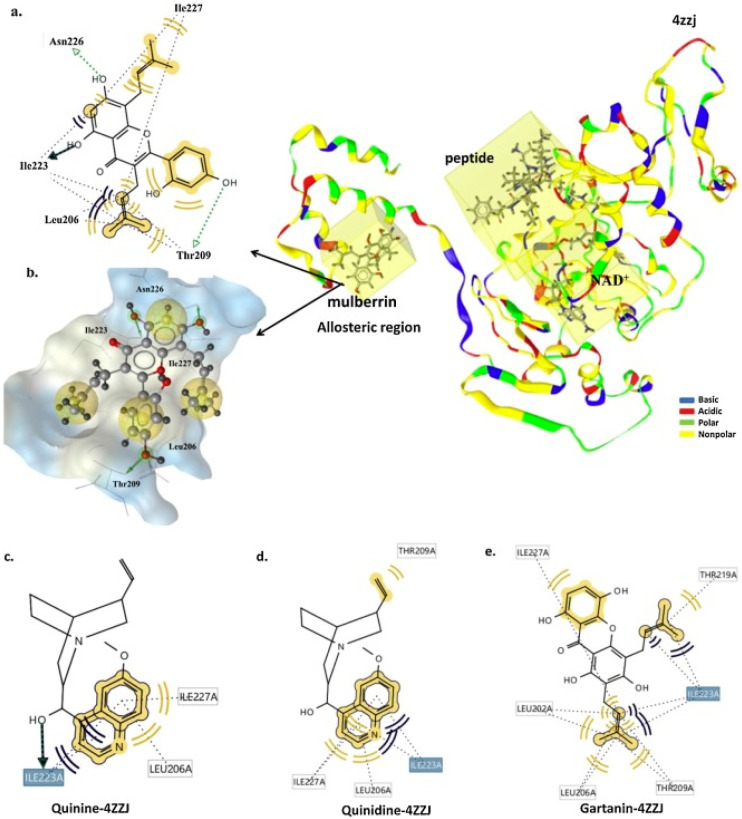
(**a**) Two-dimensional scheme of the interaction between mulberrin and the 4ZZJ complex, generated by LigandScout 4.2. (**b**) The structure of mulberrin and 4ZZJ complex shown as a mesh surface image, generated by LigandScout (hydrophilic, blue; hydrophobic, grey). The interaction between (**c**) quinine, (**d**) quinidine, and (**e**) gartanin, with the 4ZZJ [125]. Figure reproduced by permission.

Azminah et al. [125] recently combined in silico and in vitro methods to explore medicinal plant-based compounds from Indonesian Herbal Database (HerbalDB), containing 1377 plant compounds, [126,127] with the goal to and identify SIRT1 activators [126]. Pharmacophore models were generated using co-crystallized ligands that allosterically bind to the SIRT1 target (PDB ID: 4ZZJ), followed by virtual screening of HerbalDB, leading to the identification of hits, which were scored as the pharmacophore fit score using LigandScout. The top hits were subjected to molecular docking and 50 ns molecular dynamics simulation and the binding interactions were analyzed using MM-GB(PB)SA methods. These were further tested in an in vitro study using Promega implemented with SIRT-Glo™ (G6451) (substrate, developer, and nicotinamide) (www.promega.com) and SIRT1 active enzymes (S35-31H-05) using a SIRT-Glo™ luminescence assay. This study showed that the flavonoid mulberrin, the xanthone gartanin, and the alkaloids quinidine, and quinine (Figure 4) were the best candidate SIRT1 activators, while molecular docking studies showed the important residues involved were Ile223 and Ile227 at the allosteric region to interact with the identified hits [125]. Meanwhile, MM-GB(PB)SA calculations confirmed that mulberrin, gartanin, quinidine, and quinine showed activity at the allosteric region, confirming their EC_50_ in vitro values are: 2.10, 1.79, 1.71, and 1.14 μM, respectively.

## 3. Therapeutic Importance of Some Naturally Occurring Sirtuin Inhibitors and Modulators 

Several structurally diverse natural products ranging from polyphenols to chromone-derived compounds, and flavonoids to chalchones, have shown to possess stimulatory or inhibitory effects on different sirtuin isoforms. Apart from preclinical [128] and mode of action studies [129,130,131] of known sirtuin inhibitors/modulators towards drug discovery, several sirtuin inhibitors and modulators from natural sources have undergone clinical trials. In this chapter, we will report the therapeutic importance of the natural products or natural NP-derived compounds that are active on Sirtuins.

Sirtuin activating compounds, STACs, are probably the most intensively studied group of compounds in both animal models and clinical trials. Resveratrol is one of the first STACs that has been reported to activate Sirt1 [132,133].

Cardiovascular diseases (CVDs) are the leading cause of death globally and the World Health Organization (WHO) estimated that about 18 million people have lost their lives from CVDs in 2016. Resveratrol and STACs were shown to have clinical benefits in many CVDs such as hypertrophy, cardiomyopathy, and endothelial dysfunction. Cardiomyopathy, which is a progressive disease of the heart muscle and can be caused by several factors ranging from systemic diseases to certain drugs. The cause can be categorized according to whether the disease process is mainly restricted to the heart, considered to be a primary (genetic, acquired, and mixed) cardiomyopathy, or if the disease occurs from the extracardiovascular system, known as a secondary cardiomyopathy [134]. A model of anthracycline cardiomyopathies showed that activation of Sirt1 by resveratrol resulted in reduced cardiac fibrosis and improved cardiac function [135]. Sulaiman et al., also showed that resveratrol upregulates sarcoplasmic calcium ATPase and improves cardiac function in mice with diabetic cardiomyopathy.

Although several pharmacological like β blockers, angiotensin-converting enzyme inhibitors, angiotensin receptor blockers, aldosterone antagonists, and nonpharmacological therapy options [136] have been introduced for the treatment of heart failure, mortality in heart failure patients remains high [137]. Activation of Sirt1 through resveratrol treatment was found to have tremendous benefits in preventing heart failure. Resveratrol could enhance the expression of AMP-activated protein kinase (AMPK) and improve cardiac function in a rat model that has heart failure produced from myocardial infarction [138]. Tanno et al., showed that Resveratrol was capable of protecting cardiomyocytes through the enhancement of manganese superoxide dismutase levels during chronic heart failure and promoted cell survival in TO-2 hamsters. Moreover, resveratrol supported animal survival in hypertension-induced heart failure as well and was suggested to be a beneficial metabolic therapy [139]. Besides, resveratrol-treated preclinical animal models revealed vascular protective effects of this polyphenol. The compound reduced the central arterial wall inflammation and stiffening that was driven by a high fat and sucrose diet [140], it also prevented myocardial damage by upregulating the endothelial growth factor (VEGF), tyrosine kinase receptor Flk-1, and nitric-oxide synthase expression. This treatment also induced new vessel growth [141], and reduced ischemia-reperfusion (I-R) injury.

In addition, resveratrol and other STACS such as SRT2104 and SRT1720, have shown promise as a treatment against metabolic disorders. Obesity, which has been associated with chronic systemic inflammation and insulin resistance that may elevate the risk of developing diabetes, hypertension, and cardiovascular disease, is one of them [142]. Studies on mice fed with a high-calorie diet revealed that resveratrol could change the physiology of these animals towards those on a standard diet, without a significant reduction in their body weight. Moreover, it could modulate known longevity pathways and extend life-span in mice consuming excess calories [143]. Similarly, the resveratrol treatment improved the energy and metabolic homeostasis of the organism, and protected mice from the development of diet-induced obesity [144]. Studies on a non-human primate model is in agreement with these findings in which resveratrol administration improved glucose tolerance and decreased an inflammatory response caused by a high-calorie diet [145]. The promising effects of resveratrol on glucose metabolism presented in preclinical animal studies has triggered more human studies to be conducted. Resveratrol supplementation has also shown to improve insulin sensitivity in older adults with impaired glucose tolerance [146] and in male patients with type 2 diabetes [147]. Moreover, it improved metabolic function and decreased inflammation markers, and produced similar effects of undergoing a calorie restriction in obese people [148]. All these preclinical and clinical studies indicate that resveratrol holds promise as a new therapeutic strategy for both cardiovascular diseases and metabolic disorders.

Piceatannol, (trans-3,4,3′,5′-tetrahydroxystilbene or 3,3′,4,5′-tetrahydroxy-trans-stilbene), is an analog and metabolite of resveratrol. As a natural stilbene, it shares similar chemical structure and is believed to exert similar pharmacological effects as resveratrol. Several cell line and ex vivo studies have shown the beneficial effects of piceatannol on the cardiovascular system. Inflammation is known to be involved in the pathogenesis of atherosclerosis and atherogenesis, leading to coronary artery heart disease, myocardial infection, and reperfusion injury [149,150,151]. Piceatannol showed anti-inflammatory effects on human pulmonary artery endothelial cells, human umbilical vein endothelial cells, and human aortic endothelial cells, confirming its preventive effects on the development and progression of atherosclerosis as well as angiogenesis [152]. Moreover, piceatannol was shown to possess a similar antioxidative activity to that of resveratrol, but has a higher free radical scavenging capacity [153]. Hung et al., showed in their animal model studies that treatment of rats with piceatannol could suppress ischemia- or ischemia-reperfusion—induced arrhythmias and reduce the cardiac infarct size produced by prolonged coronary artery occlusion [154]. Furthermore, in vitro and in vivo studies revealed the potential therapeutic effect of piceattanol in metabolic disorders [155]. This compound could decrease cholesterol, low-density lipoprotein (LDL), high-density lipoprotein (HDL), the LDL:HDL ratio, serum free fatty acids, triglycerides, serum, and blood glucose in animal models [156,157,158,159,160]. In these studies, piceattanol improved lipid handling, lowered lipid accumulation in adipocytes and the liver, reduced body weight in a dose dependent manner, and improved the impaired glucose tolerance in obese and diabetic rodents. In summary, preclinical animal studies indicated that piceattanol may hold promise as a therapy to overcome several obesity complications and maybe a phytochemical treatment for the prevention of diabetes and hypercholesterolemia. Many cell line studies also showed that piceatannol exhibits antiproliferative activity against various tumor cells, including leukemia, lymphoma, melanoma, and cancers of the breast, ovarian, prostate, bladder, colon, and liver [161]. However, further animal model and clinical studies are required to ascertain chemopreventive effects and the antiproliferative role of piceatannol in cancer.

Trans-ϵ-viniferin, which is a dimer of resveratrol has also spurred great interest as a potential therapeutic multi-target drug candidate. In their studies, Yáñez et al., presented that this natural polyphenol could inhibit the uptake of noradrenaline and 5-hydroxytryptamine (5-HT) and demonstrated an inhibitory activity on monoamine oxidase (MAO). These results indicated potential implications and benefits of trans-ϵ-viniferin for the treatment of major depression [162]. Moreover, beneficial effects of trans-ϵ-viniferin was also evaluated for one of the most common neurodegenerative disease, Alzheimer’s disease (AD). AD is a chronic and progressive syndrome in which cognitive function deteriorates irreversibly. AD is accepted as the most common form of dementia and WHO estimates that there are about 50 million people having dementia worldwide. Vion et al., demonstrated that trans-ϵ-viniferin could induce the disaggregation of the amyloid ß (Aß) peptide which accumulates during the progression of AD and also prevent neuroinflammation in a murine model [163]. Trans-ϵ-viniferin has been also shown to inhibit the intestinal calcium activated chloride channel which added a new pharmacological potential role of trans-ϵ-viniferin as an antisecretory therapy for rotaviral diarrhea [164].

Chromenone-derived natural products such as fisetin, orientin, quercetin, and vitexin were important natural products showing modulatory effects on different sirtuin isoforms. Like stilbeneoids, they exhibited a broad spectrum of activity ranging from senotherapeutic and antidiabetic, to antitumorogenic. A fisetin-treatment decreased kidney damage, reduced markers of oxidative stress and anxiety-related symptoms, in diabetic mice [165]. Thus, fisetin might be useful for the treatment of two complications related to diabetes, diabetic nephropathy, and anxiety symptoms. Moreover, cell line and animal model studies indicate that fisetin may reduce angiogenesis, induce apoptotic cell death and consequently suppress tumor growth [166,167,168,169]. However, anticarcinogenic effects of fisetin in different types of cancer such as lung, prostate, colon, pancreas, and melanoma still needs to be evaluated more with preclinical experiments and clinical studies in human. Fisetin was also shown to reduce senescence markers in several organs and age-related pathology in mice and consequently enhance the life span [109].

As of now, there are over 70 studies that are associated with quercetin in the ClinicalTrials.gov database (http://www.clinicaltrials.gov). Both preclinical and clinical studies demonstrated that quercetin may have beneficial physiological effects on cardiovascular diseases by lowering blood pressure and reducing the risk of ischemic heart diseases and stroke. In addition, it can also decrease oxidative stress. Quercetin has been shown to enhance the effects of other chemotherapeutic agents and could counteract resistance to drugs thereby suppressing tumor growth. Quercetin is known to improve glucose homeostasis and decrease plasma glucose, cholesterol, and triglyceride levels in diabetes. Moreover, quercetin can improve dyslipidemia, hypertension, and hyperinsulinemia, along with decreasing inflammation markers and reduce body weight in obesity. Interestingly, quercetin can also aid in treating age-related conditions such as cognitive decline [170,171,172,173,174,175,176].

The health benefits of some nature-based sirtuin inhibitors and modulators have been summarized in Table 1, including the possible modes of actions of the compounds. These show a broad range of applications, including anti-aging benefits, potential for the treatment of cardiovascular diseases, metabolic disorders, anticancer agents, etc. The health benefits of nature-based sirtuin inhibitors and modulators have been summarized in Table 1, including the possible modes of actions of the compounds. These show a broad range of applications, including anti-aging benefits, potential for the treatment of cardiovascular diseases, metabolic disorders, anticancer agents, etc.

## 4. Conclusions

Sirtuins are an important class of histone deacetylases, which are involved in NAD+-dependent deacetylation reactions. Since sirtuin activation, inhibition, and modulation are involved in several relevant metabolic reactions, related to diseases like type 2 diabetes, the aging process, and inflammation, they constitute an important drug target class. NPs are known as important sources of lead compounds. In this review, we have attempted to provide a summary of the most recent results summarizing reported NPs interacting with sirtuins. This includes the potential health benefits of naturally occurring sirtuin inhibitors and modulators, along with their known modes of action and those in clinical trials. Several natural compounds and compound classes have been shown to exhibit activities against sirtuins, including flavonoids, a tanikolide, a xanthone, resveratrol, bichalcones, and alkaloids. It is hoped that NP derivates starting from the identified lead compounds would constitute the next generation sirtuins modulators.

## Figures and Tables

**Table 1:** Potential health benefits of sirtuin inhibitors and modulators from nature.

Compound (PCID *)	Health Benefits	Mode of Action	References
Fisetin (5281614)	Antiaging and against cardiovascular disease	-Demonstrates senotherapeutic activity in mice and in human tissues. -Reduces the levels of the cyclin-dependent kinase 5 (Cdk5) activator p35 cleavage product, p25, in both control and Alzheimer’s disease brains. -Elevates the levels of p25 relative to p35, causing dysregulation of Cdk5 activity leading to neuroinflammation and neurodegeneration.	[109,177,178]
	Anticarcinogenic agent, chemopreventive/ chemotherapeutic agent against cancer	-Activates caspases-7 and -9. -Increases expression of proapoptotic protein Bak and induces its mitochondrial oligomerization. -Inhibits of Akt/mTOR signaling	[166,168,169]
	Antioxidant agent	Not well known	[167]
	Antidiabetic	Lowers methylglyoxal dependent protein glycation	[165]
Orientin (5281675)	Anti-inflammatory	-Decreases the activity of myeloperoxidase (MPO) and the production of cytokines in rats. -Inhibits the nuclear translocation of nuclear factor kappa B (NF-κB) p65, the activity of NF-κB-luciferase, and the expression of NF-κB target genes. -Attenuates experimental inflammatory bowel disease (IBD) via suppression of TLR4 and inactivation of NF-κB and MAPK pathways in rats.	[179]
	Antioxidant and Antiaging	Reduces the H_2_O_2_-induced β-galactosidase activity	[180]
	Antiviral and antibacterial agent	-Shows moderate or potent antiviral activity against Para 3 virus. -The flavonoid mixture (containing orientin, rutin, quercetin, and kaempferol) at the maximum nontoxic dose of 0.048 μg/mL was shown to fully inhibit Herpes Simplex Virus Type 2 (HSV-2) of different viral titre (1, 10, 100 TCID50) on Hep-2 cells.	[181,182,183]
	Anti-inflammatory agent	-Inhibits the high mobility group box-1 (HMGB1) protein level in lipopolysaccharide- (LPS-) induced umbilical vein endothelial cells (HUVECs) as well as the HMGB1-mediated cytoskeletal rearrangements. -Suppresses LPS-induced membrane disruption, migration of monocytes, expression of cell adhesion molecules (CAMs), and LPS-induced EPCR detaching.	[184,185,186]
	Anticancer effects	-Regulates the apoptosis-related gene expression of p53 and bcl-2. -May serve as therapeutic agents for the treatment of esophageal cancer.	[187]
	Weight loss	-Represses the accumulation of intracellular triglyceride (TG) in mouse adipocyte 3T3-L1 cells. -Decreases the mRNA levels of the genes involved in adipogenesis, lipogenesis, lipolysis, and TG synthesis, and reduces the release of glycerol. -Lowers the expression of CCAAT/enhancer binding protein (C/EBP) δ in the early stage of adipogenesis, leading to a decrease in the expression of the adipogenic master transcription factors such as peroxisome proliferator-activated receptor (PPAR) γ and C/EBP.	[188]
	Protection against bone marrow damage	Reduces chromosomal aberration cells in bone marrow	[189,190]
	Other (e.g., vasodilatation, cardioprotective, radioprotective, neuroprotective, antidepressant-like, antiadipogenesis, antinociceptive, etc., effects)	-Inhibits the protein expressions of C/EBPα and PPARγ -Reduces acetic acidinduced writhing and capsaicin- and glutamate-induced pain in mice -Orientin was shown to be 20-fold more potent than the typical painkiller, acetylsalicylic acid (aspirin), and 3.5-fold more dynamic than the common anti-inflammatory drug, indomethacin	[191]
Piceatannol (667639)	Anticancer effects	-Inhibits migration and invasion of prostate cancer cells, possibly mediated by decreased interleukin-6 signaling. -Inhibits COX-1/2 and CSN-associated kinase.	[161,192]
	Metabolic diseases	-Inhibits adipogenesis. -Blocks mitotic clonal expansion. -Inhibits insulin signaling due to non-competitive binding to insulin receptor. -Lowers lipid accumulation during late stages of differentiation. -Etc.	[155]
	Cardiovascular diseases	-Activates a nuclear receptor, peroxisome proliferator-activated receptor alpha (PPAR-α) isoform, on rat hepatoma (H4IIEC3 cells) in vitro. -Lowers lipid and lipoprotein in vivo. -Etc.	[152,193]
Quercetin (5280343)	Cancer treatment	Tyrosine kinase inhibition in vivo	[171,172]
	Treatment of colitis and gastric ulcer	Not well known	[194]
	Treatment of respiratory tract infection	Not well known	[195]
	Treatment of type 2 diabetes	Was shown to be beneficial in improving the antioxidant status of patients with type 2 diabetes while having no other significant effect on glycemic control and lipid profile	[173,174,175]
	Treatment of high blood pressure	-Mode of action still under investigation	[176]
	Treatment of oral lichen planus	-Restriction of cyto-kines including IL12, INFγ, INF α, IL8, cyclooxi-genase 2 and prostaglandin E	[196]
	Other health benefits	Not well known	[170]
Resveratrol (445154)	Obesity treatment	Sirtuin modulation	[197]
	Colon cancer prevention	Inhibits a key signaling pathway involved in colon cancer initiation, the Wnt pathway, in vitro.	[198]
	Cardioprotection	Decreases low-density lipoprotein (LDL) oxidation, and functions as a direct free radical scavenger.	[199]
*trans*-(−)-ϵ-Viniferin (5315232)	Anti-inflammatory, antioxidant, platelet antiaggregatory and anticarcinogenic properties	Monoamine oxidase activity	[162]
	Prevention of rotaviral diarrhea	Inhibition of the intestinal calcium-activated chloride channel	[164]
	Potential for the treatment of Alzheimer’s disease.	Induces the disaggregation of amyloid β (Aβ) peptide	[163]
	Potential for the treatment of diabetes.	Inhibition of high glucose-induced apoptosis by maintaining Ca^2+^ and preserving mitochondrial membrane potential (MMP) levels.	[200]
Vitexin (5280441)	Anticancer effects	-Regulates the apoptosis-related gene expression of p53 and bcl-2 -May serve as therapeutic agents for the treatment of esophageal cancer -Targeting cell apoptosis in U937 cells	[187,201]
	Anti-oxidant effects	Subdues oxygen free radical and protecting the antioxidant enzyme activity in cells and the sulfhydryl in the red cell membrane protein.	[202,203]
	Lifespan extending and stress resistant properties	Reduces intracellular reactive oxygen species (ROS) accumulation in a dose-dependent manner	[204]
	Anti-inflammatory effects	-Inhibit IL-1β, IL-6, IL-8, IL-17, and IL-33 -Inhibit tumor necrosis factor-α (TNF-α) secretion -Inhibit COX-2 -Inhibit NF-κB activation -Inhibit iNOS (inducible nitric oxide synthase) -Inhibit NO, PGE2, monocyte chemoattractant protein-1 (MCP-1), and neutrophil influx -Increase in IL-10 and reduce the expression of p-p38, p-ERK and p-JNK.	[105,205,206,207,208,209,210,211]
	Anti-neoplastic effects	-Promotes autophagy through the up-regulation of Hsp90 expression and subsequent activation of endoplasmic reticulum (ER)-stress -Hsp90 may be involved in a new signaling pathway with anti-neoplastic effects of vitexin	[212]
	Protective effects against neurological and psychiatric diseases (e.g., hypoxia and ischemia injury, Alzheimer’s disease, learning, cognition and depression, Anti-nociceptive activity, Other neurological and psychiatric disorders, etc.)	-Helps to maintain blood-brain barrier (BBB) integrity and attenuate brain oedema with down-regulated HIF1-α and VEGF -Contributes to the protection against cerebral I/R injury, and it up-regulated p-ERK1/2, down-regulated p-JNK and p-P38, as well as increased Bcl-2 and decreased Bax expression in the cortex and hippocampus. -As a mediator of Aβ toxicity, ubiquitin- ligating enzyme E2-25 K/Hip-2 plays a role in the pathogenesis of AD	[129,201,213,214,215]
	Protective activity in cardiovascular system	-Inhibits the isoproterenol-induced increase in resting intracellular free calcium as well as expression of the calcium downstream effectors calcineurin- NFATc3 and phosphorylated calmodulin kinase II (CaMKII) both in vitro and in vivo -Vitexin-containing lime leaf significantly inhibited platelet aggregation in a concentration-dependent manner	[216,217]
	Protective effects against endocrine and metabolic disease (e.g., diabetes, obesity, other endocrine and metabolic diseases, anti-thyroid effect, etc.)	-Reduces postprandial blood glucose both in sucrose loaded normoglycemic mice and sucrose induced diabetic rats -Improves adipocyte functionality related to regulating PPARγ, carnitine palmitoyltransferase-1 and enzymes involved in lipogenesis -Inhibits thyroid peroxidase (TPO) activity -Inhibits the activities of P450 17α-hydroxylase/17 (CYP17A1), deoxycortisol conversion by P450 11β-hydroxylase (CYP11B1), but not P450 21-hydroxylase (CYP21A2)	[218,219,220,221]
	Anti-microbial and anti-viral effects	-Activity against anti-*Helicobacter pylori* effect is probably due to its activities on anti-MPO enzyme and inhibition of H+, K+-ATPase activity -Vitexin also exerts anti-phytoviral activity against Tobacco mosaic virus (TMV)	[222,223]

* PubChem compound ID [224].
